# The impact of positron emission tomography on primary tumour delineation and dosimetric outcome in intensity modulated radiotherapy of early T-stage nasopharyngeal carcinoma

**DOI:** 10.1186/s13014-016-0685-8

**Published:** 2016-08-24

**Authors:** Vincent W. C. Wu, Wan-shun Leung, Kwun-lam Wong, Ying-kit Chan, Wing-lam Law, Wing-kwan Leung, Yat-long Yu

**Affiliations:** 1 Department of Health Technology & Informatics, Hong Kong Polytechnic University, Hung Hom, Hong Kong; 2 Oncology Department, Princess Margaret Hospital, Kwai Chung, Hong Kong

## Abstract

**Background:**

In intensity modulated radiotherapy (IMRT) of nasopharyngeal carcinoma (NPC), accurate delineation of the gross tumour volume (GTV) is important. Image registration of CT and MRI has been routinely used in treatment planning. With recent development of positron emission tomography (PET), the aims of this study were to evaluate the impact of PET on GTV delineation and dosimetric outcome in IMRT of early stage NPC patients.

**Methods:**

Twenty NPC patients with T1 or T2 disease treated by IMRT were recruited. For each patient, 2 sets of NP GTVs were delineated separately, in which one set was performed using CT and MRI registration only (GTV_CM_), while the other set was carried out using PET, CT and MRI information (GTV_CMP_). A 9-field IMRT plan was computed based on the target volumes generated from CT and MRI (PTV_CM_). To assess the geometric difference between the GTV_CM_ and GTV_CMP_, GTV volumes and DICE similarity coefficient (DSC), which measured the geometrical similarity between the two GTVs, were recorded. To evaluate the dosimetric impact, the D_max_, D_min_, D_mean_ and D_95_ of PTVs were obtained from their dose volume histograms generated by the treatment planning system.

**Results:**

The overall mean volume of GTV_CMP_ was greater than GTV_CM_ by 4.4 %, in which GTV_CMP_ was slightly greater in the T1 group but lower in the T2 group. The mean DSC of the whole group was 0.79 ± 0.05. Similar mean DSC values were also obtained from the T1 and T2 groups separately. The dosimetric parameters of PTV_CM_ fulfilled the planning requirements. When applying this plan to the PTV_CMP_, the average D_min_ (56.9 Gy) and D_95_ (68.6 Gy) of PTV_CMP_ failed to meet the dose requirements and demonstrated significant differences from the PTV_CM_ (*p* = 0.001 and 0.016 respectively), whereas the doses to GTV_CMP_ did not show significant difference with the GTV_CM_.

**Conclusion:**

In IMRT of early stage NPC, PET was an important imaging modality in radiotherapy planning so as to avoid underdosing the PTV, although its effect on GTV delineation was not significant. It was recommended that PET images should be included in the treatment planning of NPC patients.

## Background

Intensity modulated radiotherapy (IMRT) is now routinely used to treat nasopharyngeal carcinoma (NPC) because of its better dose conformity and steeper dose gradient at the boundary of target volume. Several dosimetric studies have proven that IMRT could improve the dose coverage to the target volume with relatively lower dose to the organs at risk (OARs) [1, 2]. Accurate delineation of the gross tumour volume (GTV) becomes more important in order to deliver effective dose coverage to the tumour [3, 4]. It is because a slight discrepancy in target delineation may cause underdose of the target volume and/or overdose of the adjacent normal tissues leading to uncontrolled primary tumour or unexpected toxicities [5, 6]. To improve the accuracy of target delineation, combining the findings from different imaging modalities has been introduced in radiotherapy, which can provide more comprehensive information about the tumour extension. Because of this, image registration between computed tomography (CT) and magnetic resonance imaging (MRI) has been routinely performed for the target delineation in NPC cases in local oncology departments. The complementary roles of these two modalities are that CT provides the geometry and electron density of the anatomical structures for radiotherapy localization and treatment planning while MRI is good at differentiating malignant tumour from soft tissues [7]. The combination of CT and MRI in target delineation of NPC patients has been proven to be useful in defining the extension of tumour involvement [8].

Apart from these imaging modalities, positron emission tomography (PET) has recently emerged as a valuable imaging modality in oncology due to its unique functional imaging property which can detect early malignant changes of cells [9]. The current clinical applications of PET in oncology include diagnosis and staging. Despite PET images have lower spatial resolution, it has the potential to further improve target delineation when registered with CT or MRI images and some positive results of PET in tumours of the thoracic region have been reported [10–12]. For target delineation of head and neck cancers, the benefit of PET is still controversial [7, 13], especially when this modality introduces radiation dose to staff and is relatively more expensive. Therefore, the aims of this study were to evaluate the impact of PET on GTV delineation in IMRT of NPC patients and identify if there was any significant dosimetric change in the target volumes after including it in the treatment planning process.

## Methods

**Fig. 1:**
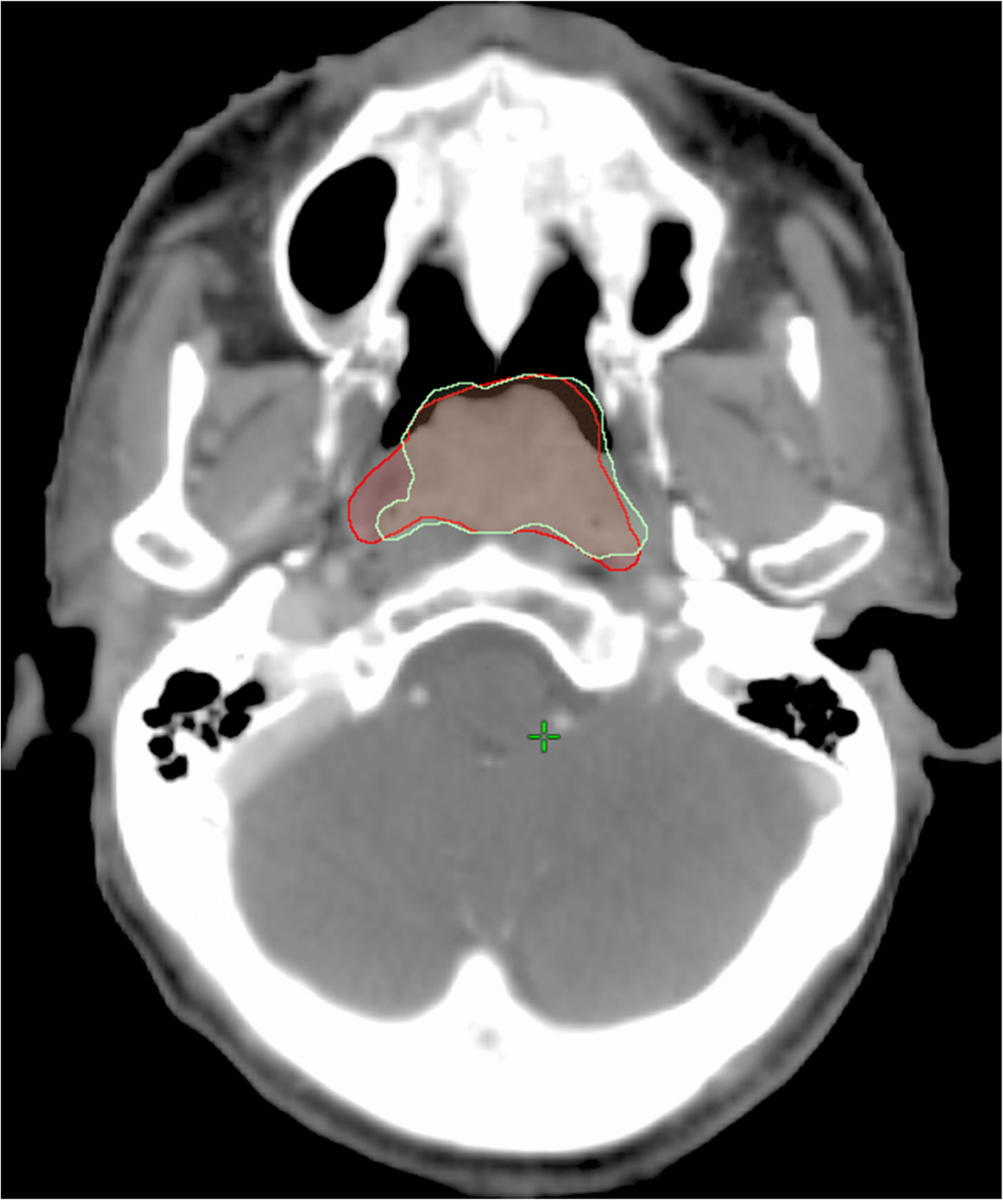
An example of difference in GTV delineation between GTV_CM_ (Red) and GTV_CMP_ (Green)

Twenty adult NPC patients with early T-stages (T1 and T2 according to AJCC 2007) treated by IMRT were retrospectively recruited. The reason for excluding high T-stages was because these tumours usually presented with bony infiltration, of which PET was known to have a poorer sensitivity [14]. Ethics approval was obtained from the local hospital and associated institution. All patients underwent imaging of the head and neck region by CT, MRI and PET. The CT and PET were taken in the same treatment position, whereas the MRI scan was carried out with the patient in normal supine head straight position. At the PET-CT unit, the patient was injected with ^18^F-FDG about 45 min before the scan. Immobilisation was applied using the tailor-made thermoplastic shell. After taking the topogram, which helped to define the scanning range from the vertex to the upper chest level, CT acquisition with slice thickness of 2.5 mm was performed. This was then followed by the PET acquisition which covered the same scan range of CT. The GTV delineation based on all the three imaging modalities (GTV_CMP_) was carried out following the protocol of local department which was referenced from the International Commission on Radiation Units and Measurement Reports 50 and 62 guidelines [15, 16]. Registration of CT and PET images were first conducted, and the result was then co-registered with the MRI through rigid registration using the MIM software (Version6.3.4, MIM Software Inc., US). The final GTV was then delineated on the CT images based on the information obtained from the PET-MRI-CT registration. Another GTV was delineated using registered images of CT and MRI only (GTV_CM_) which was performed using the same MIM software based on the same target delineation protocol by a different clinician who was blinded to the GTV_CMP_. An example of GTV delineation by the two methods is shown in Fig. 1. A routine IMRT treatment which consisted of 9 equal spaced IM beams with 6 MV and dynamic multileaf collimators (MLC) was then computed using GTV_CM_ as the only target with the GTV_CMP_ and PTV_CMP_ being switched off. 70 Gy in 35 fractions was prescribed to the PTV which was created by adding 1 cm to the GTV. PTV_CM_ was created from GTV_CM_ while PTV_CMP_ was created from GTV_CMP_. The dose requirements of the target volume were that at least 70 Gy should cover 95 % of PTV and 100 % of GTV. The organs at risks (OARs) were delineated and planning organs at risk volume (PRVs) were created for the more critical structures such as brain stem, spinal cord and optic chiasm by adding 1 mm in all directions, The planning objectives and dose constraints of the targets and OARs were based on the local planning protocol which was referenced from the Radiation Therapy Oncology Group (RTOG) 0225 protocol.

For each patient, the GTV_CMP_ and GTV_CM_ volumes were recorded. DICE similarity coefficient (DSC), which was generated by the MIM software, was used to evaluate the geometrical difference between GTV_CMP_ and GTV_CM_. DSC was defined by (V_p_, V_1_) = 2|V_p_∩V_1_|/|V_p_| + |V_1_|_,_ where V_p_ and V_1_ were the volumes of GTV_CMP_ and GTV_CM_ respectively and ∩ was the intersection. A value of 1.0 would indicate perfect volume match between the two GTVs, whereas 0 would imply no overlapping of volume exist. For dosimetric analysis of the target volume, PTV was the main focus apart from GTV because PTV dose would be more clinically relevant and expected to demonstrate more obvious difference between the two delineation methods. The maximum (D_max_), minimum (D_min_), mean doses (D_mean_), and dose received by 95 % volume (D_95_) of PTV; and D_max_ D_min_, and D_mean_ of GTV were obtained from the their respective dose volume histograms. The average values of volumetric and dosimetric parameters from each delineation method were calculated for comparison. Furthermore, the difference in GTV geometry between T1 and T2 patient groups were also evaluated. All data were tested for the normality by Shapiro-Wilk test. Paired t-test or Wilcoxon signed-rank test was used to compare the differences between the two delineation methods depending on the normality of data, whereas Mann–Whitney U test was used to compare the differences between T1 and T2 patient groups. All statistical tests were performed using Statistical Package for Social Science (SPSS) version 22 (IBM).

## Results

Sixty percent of patients (*n* = 12) presented with T-1 stage and 40 % of them were T2 (Table 1). The mean GTV and PTV volumes of the T2 group were greater than those of T1 group by 55 % and 40 % respectively.

**Table 1:** Stage distribution of the sample group (*n* = 20)

Staging^a^	No (%)
T1N0M0	2 (10 %)
T1N1M0	5 (25 %)
T1N2M0	5 (25 %)
T2N0M0	3 (15 %)
T2N1M0	2 (10 %)
T2N2M0	1 (5 %)
T2N3M0	2 (10 %)

^a^American Joint Committee on Cancer 2007 staging system

With regard to the GTV geometry generated by the two delineation methods, the overall mean GTV_CMP_ was greater than GTV_CM_ (13.5 cm^3^ vs 12.9 cm^3^) and their difference did not reach statistical significance (*p* = 0.232) (Table 2). The GTV_CMP_ was slightly greater in the T1 group but lower in the T2 group. The mean DSC value of the whole group obtained by comparing the volumes of GTV_CMP_ and GTV_CM_ was 0.79 ± 0.05 (Table 3). Similar mean DSC values were also obtained from the T1 and T2 groups separately, and there was no significant difference between these two T-stage groups (*p* = 0.691).

**Table 2:** Volume comparison between GTV_CM_ and GTV_CMP_

	GTV_CM_ (cm^3^)	GTV_CMP_ (cm^3^)	Wilcoxon signed-rank test
Group	Mean ± SD	Mean ± SD	*p* value
T1 (*n* = 12)	9.8 ± 3.7	11.5 ± 5.5	0.329
T2 (*n* = 8)	21.9 ± 8.7	19.4 ± 4.9	0.591
All (*n* = 20)	12.9 ± 7.7	13.5 ± 6.5	0.232

**Table 3:** DICE similarity coefficient (DSC) results by comparing GTV_CM_ and GTV_CMP_

	DSC	Mann–Whitney U test
Group	Mean ± SD	*p* value
T1 (*n* = 12)	0.79 ± 0.05	0.691
T2 (*n* = 8)	0.78 ± 0.04	
All (*n* = 20)	0.79 ± 0.05	

**Fig. 2:**
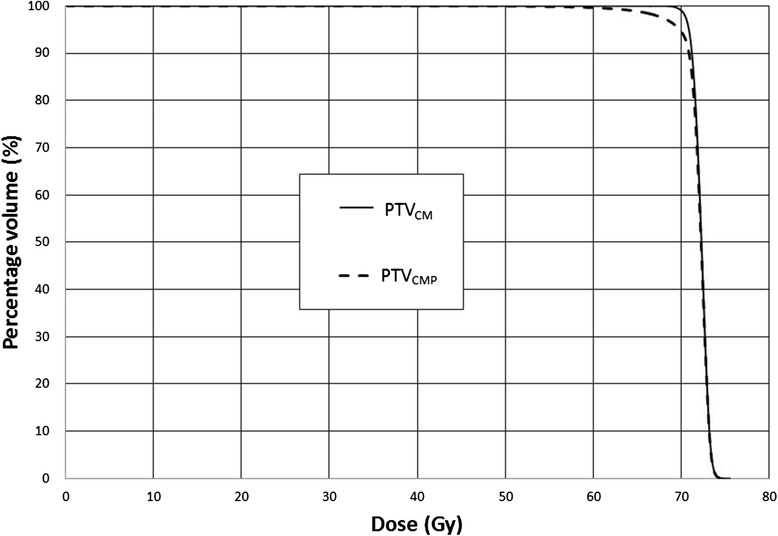
Average dose volume histograms of PTV_CMP_ and PTV_CM_

For all treatment plans, the doses to the OARs met the dose requirements as stipulated in the planning protocol. Since the plans were computed based on PTV_CM_, the dosimetric parameters of PTV_CM_ (including those of GTV_CM_) fulfilled the planning requirements, in which at least 95 % of PTV received 70 Gy, and the D_max_ and D_min_ were kept below 77 Gy and above 63 Gy respectively. When applying this plan to the PTV_CMP_, the D_min_ (56.9 Gy) and D_95_ (68.6 Gy) of PTV_CMP_ failed to meet the above-stated dose requirements and demonstrated significant differences from the PTV_CM_ (*p* = 0.001 and 0.016 respectively) (Table 4). The difference in target dose coverage between PTV_CMP_ and PTV_CM_ was also demonstrated by their average DVHs (Fig. 2). Nevertheless, the doses to GTV_CMP_ did not show significant difference with the GTV_CM_.

**Table 4:** Comparison of GTV and PTV doses between plans with and without PET in target delineation

	GTV			PTV		
	GTV_CM_	GTV_CMP_		PTV_CM_	PTV_CMP_	
	Mean ± SD	Mean ± SD	*P* value*	Mean ± SD	Mean ± SD	*P* value*
D_min_ (Gy)	70.7 ± 7.6	70.6 ± 5.7	0.895	67.9 ± 4.5	56.9 ± 6.6	0.001
D_mean_ (Gy)	72.2 ± 2.9	72.2 ± 3.7	0.796	72.2 ± 2.6	71.9 ± 1.2	0.369
D_max_ (Gy)	73.7 ± 2.2	73.7 ± 1.3	0.981	74.5 ± 2.9	74.4 ± 0.9	0.548
D_95_ (Gy)	--	--	--	71.1 ± 2.1	68.6 ± 2.6	0.016

*Wilcoxon signed-rank test

## Discussion

As far as volumetric analysis of the GTV was concerned, the current study revealed that there was only mild impact of PET on GTV delineation for the early stage primary NP tumour. The volume differences between GTV_CM_ and GTV_CMP_ were not significant with an overall mean difference of about 4.4 %. Previous studies on moderate to late stages (II to IV) head and neck cancers reported that there was a decrease in GTV volume delineation with PET when compared to those with either CT or MRI [17, 18]. Our study showed similar result for the T2 cases but the difference was not significant. In addition, as the overall mean DSC of GTV between the two delineation methods was close to 0.8, it indicated that the difference in GTV shape between the two delineation methods was not great. This implied that the introduction of PET did not lead to dramatic change of the GTV geometry. When comparing the results between the T1 and T2 groups, it was logical to see that the average GTV volume of T2 group was significant larger than that of the T1 group due to the relatively more extensive tumour invasion. However, when analysing the impact of PET on these two T-stage groups, they did not showed any obvious difference, which was reflected by the GTV volume and DSC comparisons. Since PET was a functional imaging tool and effective in detecting early malignant changes, it was able to pick up microscopic spread around the primary tumour which might not be detected by MRI or CT. Taking reference from other tumours [19–21], for early stage of NPC, the primary tumour was relatively small and the chance of extensive microscopic spread was lower compared to more advanced tumours. The contribution by PET to provide extra tumour information would not be much, and could be one of the reasons that resulted in a relatively mild change of GTV geometry.

**Fig. 3:**
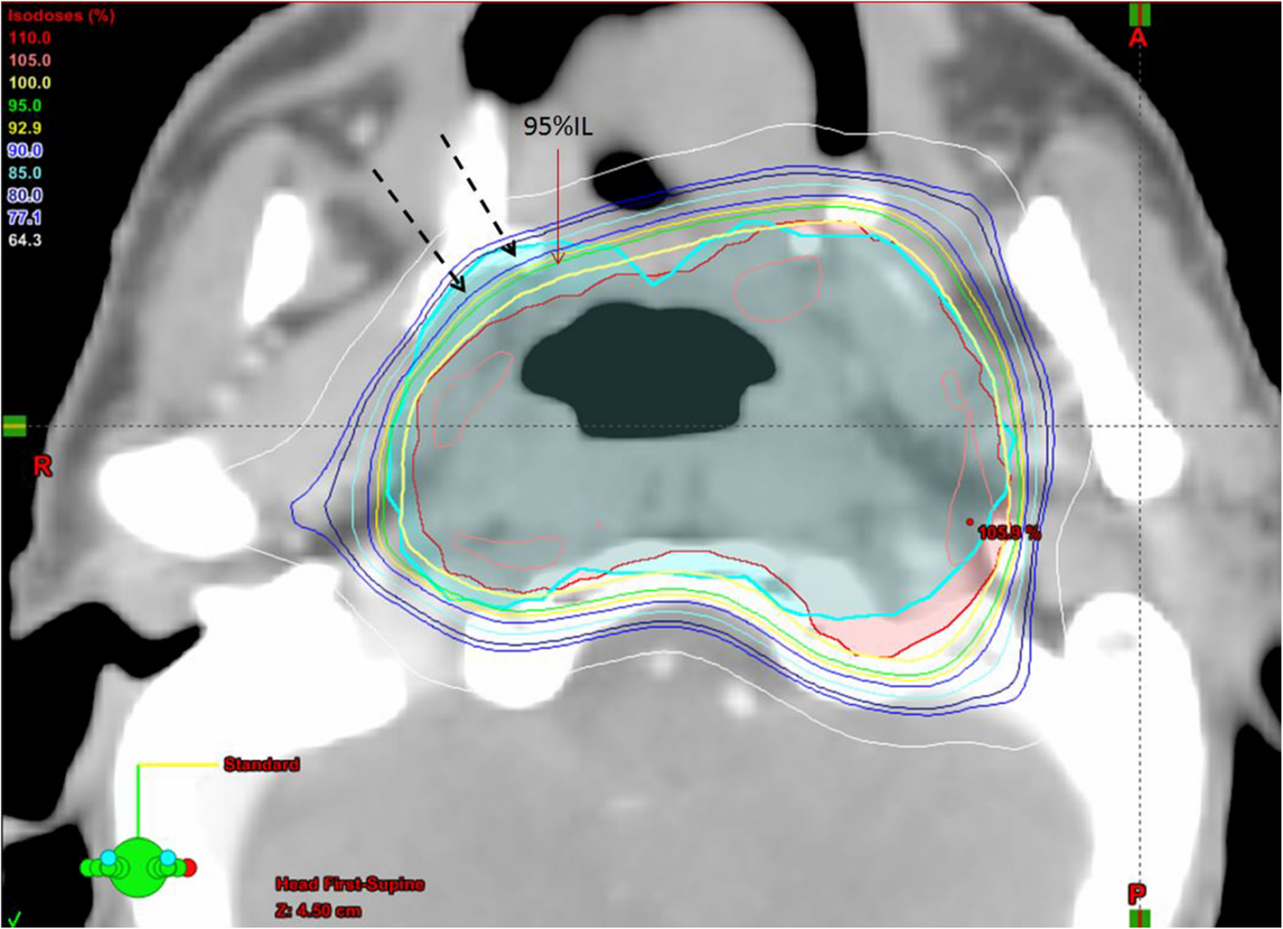
An example of IMRT plan showing the dose distribution at one of the CT slices. The plan was computed based on the PTVCM (Red). The black arrows indicate the regions of PTV_CMP_ (Cyan) receiving less than 95 % isodose level (indicated by the red arrow)

Unlike the volumetric analysis results, the dosimetric impact of PET was more significant, especially on the PTV dose. Despite the GTV doses did not show significant difference between the two delineation methods, the D_min_ and D_95_ of PTV_CMP_ were significantly lower than the PTV_CM_ and did not meet the dose requirement. This implied that there were distinct underdose regions in the PTV_CMP_ and might not be acceptable clinically. The reason for such difference in PTV coverage was because the discrepancy in target delineation was magnified in the PTV which was expanded from the GTV. After including the PET information, considerable portion of the PTV_CMP_ was found outside the original PTV_CM_ (Fig. 3). Since the treatment plan was computed according to the PTV_CM_, these PTV_CMP_ regions outside PTV_CM_ would not be adequately covered by the prescribed dose (e.g. 95 % isodose level) and therefore might not receive adequate dose. The reason that this dosimetric difference did not appear in the GTV was because the volume of the GTVs was relatively small and so were their differences.

PET is a more expansive imaging modality that involves radiation exposure to radiographers during patient positioning. Its omission can be justified if there were no added value for radiotherapy treatment planning. In the radiotherapy of early stages of NPC, although the effect of PET on the overall GTV geometry was not significant, there was impact on the dose distribution of the PTV. Our study demonstrated the treatment plans produced just from the CT and MRI was not adequate to cover the PTV generated from CT, MRI and PET, which was believed to be the more accurately delineated target. Our result echoed the report from Graf et al. [22] who reported that PET had significant impact on stereotactic radiotherapy of malignant cranial based tumours. Though PET was reported to be less satisfactory in detecting bone infiltration, it is still worth to extend this study to the more extensive tumour so as to obtain a more comprehensive picture about the impact of PET on all NPC patients.

## Conclusion

In IMRT of early stage NPC, in addition to CT and MRI, PET was an important imaging modality in radiotherapy planning so as to avoid underdosing the PTV, although its effect on GTV delineation was not significant. It was recommended that PET images should be included in the treatment planning for this group of NPC patients.

## Abbreviations

CT: Computed tomography
CTV: Clinical target volume
D_max_: Maximum dose
D_min_: Minimum dose
DSC: DICE similarity coefficient
DVH: Dose volume histogram
GTV: Gross tumour volume
IMRT: Intensity modulated radiotherapy
MLC: Multileaf collimator
MRI: Magnetic resonance imaging
NPC: Nasopharyngeal carcinoma
PET: Positron emission tomography
PRV: Planning organ at risk volume
PTV: Planning target volume
